# Experimental model of nephropathy associated with diabetes mellitus in mice

**DOI:** 10.1590/acb381123

**Published:** 2023-05-01

**Authors:** Pâmela Henrique Silva, Patrícia Henrique Silva, Adalberto Vieira Corazza, Josivaldo Godoy da Silva, Iandara Schettert Silva

**Affiliations:** 1 Universidade Federal de Mato Grosso do Sul – Programa de Pós-Graduação em Saúde e Desenvolvimento na Região CentroOeste – Campo Grande (MS), Brazil.; 2 Universidade Federal de Mato Grosso do Sul – Câmpus de Três Lagoas – Campo Grande (MS), Brazil.

**Keywords:** Kidney, Streptozotocin, Ischemia, Reperfusion

## Abstract

**Purpose:**

Nontransmissible chronic diseases, such as diabetes mellitus (DM) and nephropathy, affect a significant portion of the population, often treated due to injuries that require healing and regeneration. To create an experimental model of associated comorbidities, for healing and regeneration studies, protocols for induction of nephropathy by ischemia and reperfusion (I/R) and induction of DM by injection of streptozotocin (STZ) were associated.

**Methods:**

Sixty-four mice (*Mus musculus*), female, adult, Swiss strain, weighing approximately 20 g, were divided into four groups: G1: control (n = 24), G2: nephropathy group (N) (n = 7), G3, DM (n = 9), and G4: N+DM (n = 24). Arteriovenous stenosis (I/R) of the left kidney was performed as the first protocol. The animals received a hyperlipidemic diet for 7 days after the injection of STZ (150 mg/kg, via i.p.) and an aqueous glucose solution (10%) for 24 h. The animals in the G3 and G4 groups were observed for 14 days before receiving the diet and STZ. The evolution of nephropathy was observed using a urine test strip and the DM, through the analysis of blood glucose with a reagent strip on a digital monitor.

**Results:**

The ischemic induction protocols of nephropathy and DM with STZ, associated, were sustainable, low-cost, and without deaths. There were alterations compatible with initial renal alterations, in the first 14 days, such as increased urinary density, pH alteration, presence of glucose, proteins and leukocytes, when compared to the control group. DM was confirmed by the presence of hyperglycemia 7 days after induction and its evolution after 14 days. The animals in the G4 group showed constant weight loss when compared to the other groups. It was possible to observe morphological alterations in the kidneys submitted to I/R, regarding coloration, during surgery and after the end of the observation period, in the volume and size of the left kidney, when compared to the contralateral kidney.

**Conclusions:**

It was possible to induce nephropathy and DM associated in the same animal, in a simple way, confirmed with rapid tests, without losses, providing a basis for future studies.

## Introduction

In underdeveloped countries, diabetic nephropathy, the main glomerular disease, is recognized as one of the main causes for the appearance and evolution of chronic kidney disease (CKD). On the other hand, CKD has been shown to be one of the main causes of morbidity and mortality in diabetic patients^1^^-^ 5.

Diabetes mellitus (DM) is a chronic metabolic disorder of carbohydrates, lipids and proteins, resulting from the defective or insufficient secretory response of the hormone insulin, which results in hyperglycemia, hypoinsulinemia, ketoacidosis, polydipsia, polyuria, polyphagia and weight loss^1^.

Among the many complications of DM are microvascular changes, cardiomyopathy, encephalopathy, retinopathy, neuropathy, and nephropathy. Diabetic retinopathy leads to visual problems, risk of falls and blindness, in last cases^1^^-^ 5.

Permanent hyperglycemia promotes several metabolic alterations^6^, which, due to specific and precise molecular events, lead to diabetic nephropathy with morphological alterations arising from the synthesis of collagen, leading to wound healing complications^7^^-^ 10.

Occlusion of the renal pedicle causes the highest levels of renal histological damage, along with generalized hemorrhagic congestion. Renal vascular congestion and edema decrease on reperfusion, but tubular epithelial damage does not change significantly. Thus, there is damage to the renal tissue, due to ischemia associated with renal hemodynamic, excretory and urinary concentration dysfunctions developed proportionally. Experimental models of renal ischemia have been frequently described in the literature, in various species and lineages^1^^,^ 2 ^,^ 9 ^,^ 10. These are morphologically and cellular characterized, as well as by biochemical tests^11^.

Previous studies enabled the effective induction of the animal model of nephropathy^2^^,^ 5 ^,^ 10 ^-^ 13. Models performed in rats and rabbits have similar characteristics. The choice of mice for the present study was due to the viability of animal production and their physiological characteristics, which are strongly similar to the human biological system. These animals are still very susceptible to streptozotocin (STZ) cytotoxicity, which allows them to be more effective in inducing the diabetes model, in addition to being better in terms of handling and maintenance convenience^12^^,^ 13.

In this sense, the present experimental model, associating nephropathy, due to ischemia and reperfusion (I/R), and DM, after a high dose of STZ, opens space for several studies, since the presence of these comorbidities are associated with other pathologies and systemic alterations, such as obesity, cardiac and vascular diseases.

## Methods

### Sample and experimental groups

**Figure 1:**
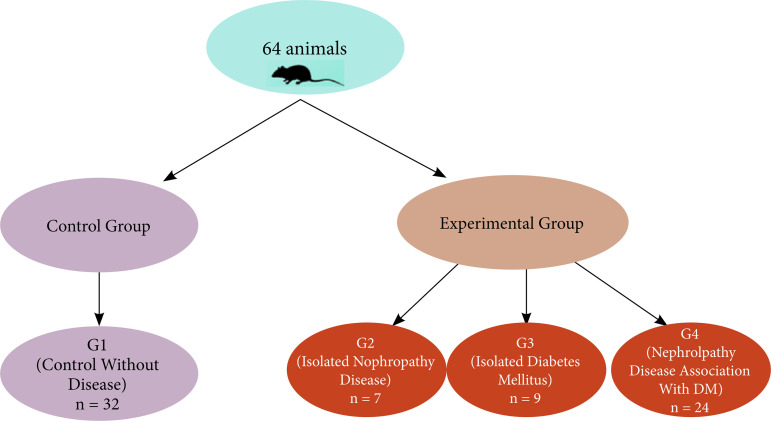
Flowchart of distribution of animals in groups and protocols. The flowchart shows the random division of animals into groups: G1 (healthy control without disease); and the experimental group subdivided into G2 (isolated nephropathy disease); G3 (isolated DM); and G4 (nephropathy disease associated with DM).

Sixty-four female and adult mice (*Mus musculus*) of the Swiss strain, weighing approximately 20 g and with mean age of 50 days, from the Central Animal House of Universidade Federal de Mato Grosso do Sul (UFMS), were used. The animals were divided into four groups: Group 1 (G1): control without disease (n = 24); Group 2 (G2): isolated nephropathy disease (n = 7); Group 3 (G3): isolated DM (n = 9); and Group 4 (G4): nephropathy disease associated with DM (n = 24), as shown in Fig. 1.

### Procedures

This study was approved by the Animal Ethics Committees of the Universidade Federal do Mato Grosso do Sul (UFMS) (Protocol CEUA/UFMS No. 1065/2019). The experiment was carried out at in the Laboratory of Experimental Models of Disease (LMED) of the Faculty of Medicine (FAMED) at UFMS.

The mice were acclimatized for 7 days, receiving a standard diet of Nuvilab CR1-Nuvital and water ad libitum. After the acclimatization phase, they were randomly distributed into four groups.

All the animals were kept in a ventilated rack with individual microisolators, in a climate-controlled room with controlled environmental conditions and a 12-h light/dark cycle.

### Surgery and ischemic induction of the nephropathy model

By means of aseptic technique, the animals were submitted to the surgical protocol of renal ischemia, after anesthesia with intraperitoneal doses of ketamine 10% (80 mg/kg) and xylazine (3 mg/kg).

The animals were submitted to a laparotomy on the left flank for access to the kidney and pedicle isolation. The renal artery and vein were clamped with atraumatic forceps isolated by a latex tube for 10 min and, during this period, ischemia was visually confirmed by changes in the organ’s color. After the forceps was released, renal reperfusion occurred, visually confirmed by the return of the organ’s initial and natural color. The kidney was repositioned and the incision closed in layers with 4-0 mononylon. All animals received analgesia and were monitored for 14 days.

Protocol monitoring in animals undergoing renal I/R surgery was performed with the 10-parameter Uri-Color Check Wama Diagnóstica test strip to detect renal alterations in a semiquantitative manner. For this purpose, the animal was manually restrained, removed from the individual microisolator and positioned on the bench, waiting for spontaneous urination and drops of urine were deposited on the test strip.

Data collected from all animals were analyzed as follows, in four stages: time 1 (T1) – before the surgical procedure on the 1st day; time 2 (T2) – 14 days after surgery; time 3 (T3) – 21 days after the procedure; and time 4 (T4) – on the 28^th^ day at the end of the experiment.

### Diabetes induction after I/R induction of nephropathy model

Fourteen days after surgery for nephropathy induction, the animals in the G4 group were prepared for the induction of the DM protocol type 1. As well as the animals of the G3 group.

Diabetes was induced by intraperitoneal (i.p.) injection of STZ (Sigma, St. Louis) administered in a single high dose of 150 mg/kg, diluted in 0.1 mol L^–1^ citrate buffer (pH 4.5). After 2 h of the induction period, they began to receive a hyperlipidemic Rhoster (Nuvilab, Brazil) diet for 7 days, with water being replaced by an aqueous glucose solution (10%) for 24 h. After this period, they received a standard diet and water *ad libitum*.

The monitoring of glycemic levels to confirm the diabetic condition was carried out by measuring blood glucose in four stages: time 1 (T1) – before I/R induction; time 2 (T2) – 14 days after nephropathy induction surgery; time 3 (T3) – 21 days after the surgical procedure and the 7^th^ day of DM induction; and time 4 (T4) – on the 28^th^ day after surgical induction and the 14^th^ day of DM induction, at the end of the experiment. Animals with glycemic values greater than or equal to 200 mg/dLof blood were considered diabetic.

Glycemic levels were analyzed using an Accu-Chek Active digital blood glucose monitor, through puncture of the tail and perforation of the caudal vein with a hypodermic needle, and depositing a drop of blood on the reagent strip of the blood glucose monitor.

The period for establishing the nephropathy model was 14 days and after another 14 days the DM model was established.

Groups G1 and G2 received a species-specific balanced commercial diet Nuvilab CR1-Nuvital. The G3 and G4 groups received the same diet as the G1 and G2 groups during the ischemic nephropathy induction procedures. Seven days after the surgery, the animals in the G3 and G4 groups received a hyperlipidemic Rhoster diet, which was maintained for another 7 days after D induction, after receiving STZ. All animals had access to water ad libitum.

The animals were weighed during the experimental period following a four-step scheme. As well as the measurement of serum blood glucose levels in a digital glucometer after installing associated protocols.

The protocol lasted 28 days until irreversible hyperglycemia was observed.

### Euthanasia

A combination of xylazine (20 mg/kg) and ketamine (20 mg/kg) was administered i.p. in a lethal dose for the euthanasia of the animals.

### Statistical analysis

All data were tabulated and expressed as mean ± standard deviation (SD). The variables body weight, glycemic levels, glucose, urobilinogen, pH, density and protein were statistically tested using multivariate analysis of variance, using the Kruskal–Wallis test, with a significance level set at p ≤ 0.05, using the BioEstat 5.3 program.

The other data were presented according to the presence, absence or normality established according to the rapid biochemical test.

## Results

The ischemic induction protocols of nephropathy and DM with STZ associated were sustainable, of low cost, and there was no death of any animal.

**Figure 2:**
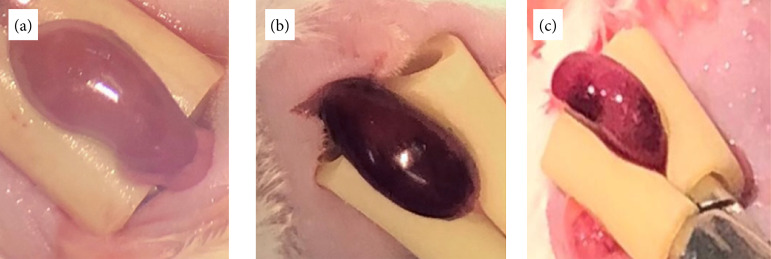
Photograph showing in **(a)** normal coloration at the onset of ischemia; in **(b)** dark staining of the kidney at 10 min of pedicle occlusion, and **(c)** shows the kidney in the process of reperfusion after releasing the renal pedicle.

Morphological alterations were observed in the kidneys submitted to I/R, regarding staining, during surgery and after the end of the observation period, in the volume and size of the left kidney, when compared to the contralateral kidney, as shown in Fig. 2.

These changes are consistent with early renal changes within the first 14 days. The detection of alterations in the urinary tract was performed using test strips for urinary analysis, in a semi-quantitative manner, with the 10-parameter Uri-Color Check Wama Diagnóstica. Statistically significant changes in pH were observed when comparing groups G1, G2, G3 and G4 at times T2, T3 and T4, having as significance level p ≤ 0.05, as well as density, protein, urobilinogen and urinary glucose, as well as the presence of proteins and leukocytes in animals from groups G2, G3 and G4 when compared to group G1. The animals in G1 presented normality parameters during the analysis, as shown in Table 1.

**Table 1:** Glycemic levels, Body Weight and Values of urine analysis parameters.

Groups	Time	T1 1^st^ day	T2 14^th^ day	T3 21^st^ day	T4 28^th^ day
	Parameters				
G1	Glycemic levels	121.1 ± 15.9	132.5 ± 16.1	145.3 ± 17.9	150.3 ± 16.8
	Body weight	25.9 ± 1.8**	27.5 ± 1.3	28.9 ± 1.4	29.7 ± 1.0
	Urobilinogen	N	N	N	N
	Glucose	-	-	-	-
	Ketones bodies	-	-	-	-
	Bilirubin	-	-	-	-
	Protein	-	-	-	-
	Nitrite	-	-	-	-
	pH	8.4 ± 0.5**	8.4 ± 0.5	8.5 ± 0.5	8.5 ± 0.5
	Blood/Hemoglobin	-	-	-	-
	Density	1028.8 ± 2.7**	1027.9 ± 4.1	1029.0 ± 2.5	1028.5 ± 3.5
	Leucocyte	-	-	-	-
	Glycemic levels	134.1 ± 15.3	119.9 ± 16.5	131.3 ± 14.4	149.0 ± 10.8
	Body weight	25.7 ± 1.1**	27.7 ± 1.4	27.0 ± 1.4*	26.3 ± 1.6*
	Urobilinogen	N	1.0 ± 0.0*	2.0 ± 0.0*	2.6 ± 1.4*
G2	Glucose	-	42.9 ± 53.5*	71.4 ± 48.8	85.7 ± 37.8
	Ketones bodies	-	-	-	-
	Bilirubin	-	-	-	-
	Protein	-	37.1 ± 45.0	41.4 ± 42.2	45.7 ± 38.7
	Nitrite	-	+	+	+
	pH	8.3 ± 0.5**	7.3 ± 0.3*	7.1 ± 0.2*	6.5 ± 0.4*
	Blood/Hemoglobin	-	-	-	-
	Density	1029.3 ± 1.9**	1022.9 ± 2.7	1012.9 ± 2.7*	1006.4 ± 2.4*
	Leucocyte	-	-	+	+
G3	Glycemic levels	166.8 ± 26.9	394.2 ± 52.1	393.6 ± 75.2	361.0 ± 67.9
	Body weight	27.7 ± 1.6**	29.4 ± 3.1	31.9 ± 4.4*	28.8 ± 4.2
	Urobilinogen	N	1.1 ± 0.3*	1.3 ± 0.5*	1.4 ± 0.5*
	Glucose	-	77.8 ± 44.1*	183.3 ± 79.1*	277.8 ± 83.3*
	Ketones bodies	-	+	+	+
	Bilirubin	-	-	-	-
	Protein	-	83.3 ± 88.0*	121.1 ± 106.5*	158.9 ± 108.2*
	Nitrite	-	+	+	+
	pH	8.7 ± 0.5**	7.6 ± 0.5*	7.2 ± 0.3*	6.7 ± 0.3*
	Blood/Hemoglobin	-	-	-	-
	Density	1028.9 ± 2.2**	1016.7 ± 3.5*	1010.0 ± 4.3*	1006.1 ± 2.2*
	Leucocyte	-	+	+	+
G4	Glycemic levels	139.7 ± 16.0	237.8 ± 17.4	314.7 ± 26.7	371.7 ± 25.8
	Body weight	26.1 ± 1.8**	24.9 ± 1.1*	27.3 ± 1.7*	27.3 ± 1.4*
	Urobilinogen	N	1.5 ± 0.5*	2.3 ± 1.3*	3.4 ± 2.4*
	Glucose	-	150.0 ± 72.2*	241.7 ± 136.5*	316.7 ± 168.5*
	Ketones bodies	-	+	+	+
	Bilirubin	-	-	-	-
	Protein	-	70.8 ± 35.3*	168.8 ± 116.3*	183.3 ± 100.7*
	Nitrite	-	+	+	+
	pH	8.5 ± 0.5**	7.6 ± 0.4*	6.9 ± 0.2*	6.2 ± 0.2*
	Blood/Hemoglobin	-	-	-	-
	Density	1028.1 ± 3.2**	1020.0 ± 3.9*	1011.0 ± 3.9*	1006.7 ± 2.4*
	Leucocyte	-	+	+	+
Kruskal-Wallis		≤ 0.05	≤ 0.05	≤ 0.05	≤ 0.05

Results are presented as mean ± SD of mean.

(*) statistically significant.

(**) statistically not significant.

(N) within the normal range. (+) positive or present. (-) negative or absent. By the analysis of variance of the Kruskal–Wallis test between the groups in the analyzed times considering the significance level of p ≤ 0.05.

Regarding pH and density, animals from groups G2, G3 and G4 were statistically smaller when compared to group G1 at times T2, T3 and T4. Identical results were observed when analyzing urobilinogen. At T2 time, the density, protein and glucose of the animals in the G1 group were within normal limits, while the animals in the G2, G3 and G4 groups showed significant changes with a significance level of p ≤ 0.05. At the other analyzed times (T3 and T4), there was a statistically significant difference in the G3 and G4 groups regarding the investigated urine markers (protein and glucose).

The DM condition was confirmed by the presence of hyperglycemia after the 7^th^ and 14^th^ day of the disease induction protocol. After 7 days of the DM induction protocol in the animals, in all induced animals of both groups G3 and G4, there was an increase in glycemic levels characterizing the disease. There was a statistically significant difference between groups when compared at the evaluated moments, with a significance level of p ≤ 0.05, as observed in Table 1.

Throughout the experiment, the animals were weighed. The animals from G1 had higher average body weight throughout the experiment when compared to G4 with significance of p ≤ 0.05 at times T2, T3 and T4 when compared to T1. There were significant differences at moments 2, 3 and 4 when comparing the G4 group to the other groups G1, G2, G3, which presented a significantly lower mean body weight than the other groups. These data were expressed as mean and standard deviation, as shown in Table 1.

## Discussion

These animal disease models, induced in this experiment in mice, concomitantly presented all the characterization results similar to the models already demonstrated in rats (nephropathy and DM) as well as in rabbits with nephropathy^11^^-^ 17. The first experiment linking nephropathy and diabetes dates from 2003, when the condition was studied in obese diabetic gene (*db/db*) of mice^18^. Studies have shown that mice are more susceptible to the toxic effects of STZ and that this animal model is similar and allows a reliable recapitulation of the morphological alteration of human diabetic nephropathy^5^^,^ 10 ^,^ 18 ^-^ 20.

Recently, several authors have reported that vascular occlusion of the kidney, unilaterally for 10 min^12^ or bilaterally for 30 min^11^, leads to morphological alterations triggered by reperfusion injuries of inflammatory or cytotoxic origin, even if due to vascular complications and/or aggravated by diabetes^7^. In addition, proteinuria occurs when there is kidney damage, can be identified by reagent strips, and is part of the characteristics that classify CKD, associated or not with other symptoms, which in more advanced stages also include weight loss and dehydration^19^.

Laboratory tests are essential for carrying out laboratory analysis of urine, being an important non-invasive test for several kidney pathologies. In addition to assisting in the diagnosis, treatment and follow-up of pathologies that may progress to renal failure^21^^,^ 22.

Urinalysis through semiquantitative biochemical analysis is a simple, quick and safe procedure that can provide valuable information about the function of the urinary system, as well as other organs and body systems. These findings make it possible to identify several pathologies, from urinary tract infections to even systemic pathologies, such as DM. The procedure is simple, disregarding the need for anesthesia and other invasive procedures, similar to that performed in mammals, in which it is possible to collect urine free or by manual expression, which makes it the exam of choice, since catheterization is impracticable for smaller rodent species, such as mice and hamsters. The rodents have a more alkaline normal urine pH ranging from 8 to 9, data compatible with what was observed in the G1 group throughout the experiment^23^.

Urinary pH values lower than 8 are associated with high protein diets, catabolic states, hunger and fever. According to the literature, healthy animals may have small amounts of urinary glucose in acute or chronic painful situations. In experimental models of DM, levels of glucose in the urine between 300 and 600 mg/dL were shown, values similar to those found in this study in the study groups G3 and G4, confirming the pathological state and the DM condition of the animals^23^.

Another important and characteristic clinical sign of diabetes is the presence of ketone bodies in the urine. As for urinary bilirubin, increased values are correlated with the destruction of blood cells or even muscle, a situation commonly associated with chronic systemic conditions, such as DM and CKD^23^.

The test strips are also reliable and safe for detecting blood in the urine. Under normal conditions, rodent urine has very few red blood cells (0-3 cells/high-power field) or none at all. In pathological conditions, in the face of an inflammatory condition or damage to the urinary system, there is a significant increase in the count of red blood cells present in the urine. In this study, during the experimental period, hematuria was not observed in the studied groups (G1, G2, G3 and G4)^23^.

Eventually, the high presence of protein in the urine is an important indicator of kidney disease, preceding clinical symptoms. Proteinuria is a renal marker that constitutes an important independent risk for renal prognosis. Proteinuria allows the early detection of kidney damage, in situations where there have not yet been changes in serum creatinine levels, expressing a risk factor for endothelial dysfunction and injury^21^^,^ 22.

Low molecular weight proteins and urinary enzymes are used as biomarkers of tubular injury to detect small changes in the function of tubular epithelial cells, which occur early and before changes in classic renal markers, such as plasma creatinine^21^^,^ 22. These findings corroborate the results obtained in the present study, since there was a significant increase in urinary protein levels in groups G2, G3 and G4, mainly confirming the pathological state caused by nephropathy, DM and their association.

Administration of a single dose of injectable STZ (150 mg/kg, i.p.) after a 12-h fast, followed by a hyperlipidemic Rhoster diet for 7 days and an aqueous glucose solution (10%) for 24 h, allowed reproducing the model of DM, after 7 days of induction in the animals, in all 33 animals induced. There was a significant difference between the groups when compared in the evaluated times, as shown in Table 1. This finding corroborates the results obtained by He et al.^24^, where after in a single dose of 120 mg/kg of STZ, i.p., 17 of the 20 animals submitted to the procedure presented increased glycemic levels, being considered diabetics. In an experimental study, Sudirman et al.^25^ used a high dose of STZ (150 mg/kg body weight, single dose) for the induction of type 1 DM followed by 12 h of supply of an aqueous solution of glucose (10%) ad libitum, were successful, as all animals that received the i.p. presented fasting hyperglycemia and increased serum glucose levels. Tang et al.^26^, in their comparative study of renal disorders in human and animal patients, for the induction of an experimental model of diabetes associated with renal disease, used a dose of 150 mg/kg of STZ in female mice, injected in two doses via i.p.

Feeding a hyperlipidemic diet, soon after the STZ injection, aimed to prevent the reversal of the hyperglycemic condition. The offer of diets with 60% of the total calories from lipids allows the establishment of a condition of insulin resistance in rats^20^. Insulin resistance, with or without diabetes, reflects an abnormality in peripheral tissue insulin resistance, giving etiopathogenic or pathophysiological margins to different clinical situations in the population, such as fatty liver disease, nephropathy, heart failure, among others^20^.

For this study, the administration of a single dose of STZ was chosen for the induction of diabetes, in order to minimize the stress of the procedure for the animals, since intermediate to high doses have been proven, in previous studies, to be effective for the induction of the diabetes model, since they affect the beta cells of the pancreatic islets^26^.

Numerous studies have pointed to the cytotoxic effect of STZ on pancreatic beta cells, which justifies its application and efficacy in the induction of animal models of diabetes^9^^,^ 14 ^,^ 15 ^,^ 18 ^,^ 20 ^,^ 24 ^,^ 25 ^,^ 27. In this study, the choice of a single dose aimed to avoid rapid necrosis of beta cells in pancreatic islets in the short term, as shown by Sun et al.^27^.

During the experiment, the animals were weighed at four times. The animals in the associated diseases group (G4) showed constant weight loss when compared to the healthy group (G1), with a significance level of p ≤ 0.05. Weight loss can cause numerous complications, from delayed tissue repair to biochemical changes, as well as cardiomyopathies, encephalopathies, neuropathies and nephropathy^1^^,^ 3 ^,^ 25 ^,^ 27 ^,^ 28.

Currently, diabetes, recognized as a socioeconomic disease, is considered one of the main causes for the appearance of kidney diseases in developed countries. Diabetic nephropathy is the main cause of CKD in the world and can lead to death^3^^,^ 4 ^,^ 25 ^,^ 28.

Hyperglycemia causes several metabolic changes. Activation of protein C kinase increases the production of reactive oxygen species, due to overproduction of nitric oxide in the mitochondrial chain, generating oxidative stress. This process, then, gives rise to an inflammatory response, in view of the increase in free radicals, which results in ischemic injury, as there is a reduction in antioxidant defenses. In addition, the toxicity caused by hyperglycemia also leads to a decrease in the amount of glycosaminoglycans in the glomerular basement membrane, a reduction in the total number of glomeruli, mesangial expansion and thickening of the glomerular basement membrane 6 ^-^ 11 ^,^ 29.

This experiment addresses the need for low-cost disease models and the possibility of using fewer animals during induction. Thus, the nephropathy model, surgically induced by I/R, allows the animals to remain alive and able to receive protocols of diabetes induction by STZ, seeking to mimic diabetic nephropathy. Therefore, this model will allow the expansion of further studies on the complications of these pathologies, as well as their association with other comorbidities, such as heart disease, encephalopathies and chronic metabolic syndromes, obesity, among others.

## Conclusion

It was possible to induce nephropathy associated with DM in the same animal, in a simple way and with quick tests, facilitating the analysis and reducing the need for difficult collections, given the size of the animal. All the animals stay alive, presenting continuous and stable evolution of the disease during the 28-day observation period.

## Data Availability

The data will be available upon request.
